# Determinants of COVID-19 vaccine acceptance and hesitancy among healthcare professionals in the Kintampo North Municipality, Bono East Region, Ghana

**DOI:** 10.4314/gmj.v56i3.4

**Published:** 2022-09

**Authors:** Mubarick N Asumah, Abdulai Abubakari, Brian Fosu, Edem K Dzantor, Prince D Agyapong, Samuel BE Harrison, Gavin Apio, Abdul-Kahar Abukari

**Affiliations:** 1 Department of Global and International Health, School of Public Health, University for Development Studies, P.O. Box TL1350, Tamale Northern Region, Ghana; 2 Ghana Health Service, Kintampo Municipal Hospital, P.O. Box 192, Kintampo Bono East, Ghana; 3 Department of Epidemiology and Biostatistics, School of Public Health, University for Development Studies, P.O. Box TL1350, Tamale Northern Region, Ghana; 4 Kintampo Health Research Centre, Research and Development Division, Ghana Health Service P.O. Box 200, Kintampo, Bono East Region, Ghana; 5 Academic and Student's Affairs Department, University for Development Studies, P.O. Box TL1350, Tamale Northern Region, Ghana

**Keywords:** Acceptance, COVID-19, healthcare professionals, hesitancy, vaccine

## Abstract

**Objectives:**

To assess the determinants of COVID-19 vaccine acceptance and hesitation among Health Care Professionals (HCPs) in the Kintampo North Municipality of Ghana.

**Design:**

An analytical cross-sectional study.

**Setting:**

The study was carried out in the Kintampo North Municipality.

**Participants:**

All health care professionals within the Kintampo North Municipality of Ghana.

**Main outcome measure:**

Acceptance of COVID-19 vaccine.

**Results:**

In all, 215 HCPs were included in this study. The overall vaccine acceptance was 78.6% among HCPs, while 21.4% were hesitant to receive the COVID-19 vaccine. Majority (57.7%) of HCPs believed that COVID-19 vaccines were safe. The following factors were found to influence vaccine acceptance significantly; those who knew someone who has taken the vaccine (adjusted Odds Ratio [aOR]; 14.9, 95% Confidence Interval [95% CI];5.0–45.0, p<0.001), those who think COVID -19 vaccine in Ghana was safe (AOR;9.2, 95%CI;3.3–25.8, P<0.001), those who said vaccines are effective in controlling COVID-19 transmission (aOR=5.0, 95%CI;2.1–12.4, p<0.001), and those who have never refused vaccines in the past (aOR=7.8, 95CI;1.6–37.8, p=0.01).

**Conclusion:**

The study indicated high COVID-19 vaccination acceptability among HCPs. However, some HCPs are hesitant to take COVID-19 vaccinations immediately. Increased adoption of COVID-19 vaccinations among HCPs and the broader Ghanaian population requires concerted efforts, including strengthening public health education on the perceived risks and safety of COVID-19 vaccines.

**Funding:**

None declared

## Introduction

In March 2020, the Coronavirus Disease 2019 (COVID-19) was declared a pandemic, and it has since become a serious public health hazard worldwide.^1^ Following the emergence of COVID-19 disease, some safety protocols such as fumigation of all public places, social and physical distancing strategies, the mandatory wearing of masks in public spaces, and in extreme instances, total or partial lockdown are being implemented in many countries.^2^

Despite all these strategies, currently, some countries are hit by the third wave of the virus. Worldwide, 187,519,798 persons have contracted COVID-19, with 4,049,372 deaths as of July 14, 2021, with 3,400,884,367 persons so far vaccinated against the COVID-19 disease.^3^ In Ghana, by 15th May 2021, 93,456 cases had been detected with 771 deaths which included health care professionals (HCPs).^4^

Although adhering to COVID-19 safety protocols are essential to containing the virus^5^,^6^, vaccines are one of the most effective strategies for halting and preventing viral transmission.^7^,^8^ By the second month of 2021, over seventy vaccines were at various stages of development, with approximately twenty of these vaccines in phase III clinical trials.^9^ While most vaccines are progressing steadily, others have been approved and are currently being used in many countries. 10 Some vaccinations are now approved for human use and are deemed safe and effective, including AstraZeneca, Pfizer, Janssen, Johnson and Johnson, Sputnik V, Moderna, Sinovac, and Sinopharm.^11^–^13^ In Ghana, efforts are being made to procure more Oxford/AstraZeneca vaccines to vaccinate the citizenry. As of May 7, 2021, eight hundred and fifty-two thousand and forty-seven (852,047) AstraZeneca vaccine doses had been administered in Ghana.^14^ Frontline healthcare professionals have been recognised among those who received the vaccine.

Despite the overwhelming evidence on the beneficial impact of vaccines on health outcomes, vaccine hesitation among individuals and communities still exists. Vaccine hesitancy refers to a person's unwillingness, delay in accepting, or refusal to receive immunisations despite vaccine delivery services being available.^15^ It is a complex dynamic interplay of factors that vary across persons, time, location, and vaccinations and is heavily influenced by complacency, convenience, and confidence.^16^,^17^ Vaccine hesitancy has become a global challenge, with vaccine hesitation increasing steadily, while only 37% of countries are undertaking assessments of vaccine hesitancy. The World Health Organization (WHO) has emphasised that vaccine hesitancy was among the top ten global health threats in 2019.^18^ Currently, some health care professionals and the general Ghanaian population have been very sceptical about accepting to take the COVID-19 vaccine. In many low- and middle-income countries, including Ghana, there is insufficient research on the causes of vaccine hesitancy.^19^,^20^ A study on COVID-19 vaccine acceptance in Ghana found that 39.2 per cent of respondents were willing to accept the COVID-19 vaccine.^21^ Research on COVID-19 vaccine hesitancy among Ghanaian health professionals remains limited.

The fact that COVID-19 vaccinations are available does not imply that they will be accepted or used.^11^ Available literature has shown that the acceptance of the COVID-19 vaccine varies and depends on various factors such as public confidence in the COVID-19 vaccine, literacy or educational level of individuals, ethnic beliefs, myths and misconceptions about the vaccine and/or the virus. 22–25 COVID-19 vaccination acceptability was higher among research participants in 19 nations, according to a survey.^26^ However, available research shows that not all health professionals are willing to take the COVID-19 vaccine, even if the vaccines were made available. 27,28 For instance, in the USA, only 36.0% 29, in Japan, 62.1% 30, and in the Democratic Republic of Congo, only 28% of health care professionals were prepared to receive the COVID-19 vaccine.^31^ Concerns about vaccination safety, side effects, and the speed with which COVID-19 vaccines are developed and approved have been mentioned as reasons for reluctance to accept COVID-19 vaccines.^29^,^32^–^34^

Healthcare professionals are trustworthy information sources for patients.^27^ The attitude of HCP toward COVID-19 vaccines is an important factor in COVID-19 vaccine uptake among the general public.^26^ Understanding the factors underlying HCPs vaccination acceptance and refusal rates has implications for future vaccine programs for the Ghanaian population and other developing countries. It is important to conduct multiple studies on the COVID-19 vaccine in multiple settings, from which a baseline targeted intervention can be developed. The current study assessed COVID-19 vaccine acceptability among healthcare professionals (HCP) in Kintampo North Municipal, Ghana.

## Methods

### Study setting

The research was carried out at the Kintampo Municipal Hospital at Kintampo North Municipality. “The Municipality is strategically positioned in the heart of Ghana, between latitudes 8°45'N and 7°45'N, and longitudes 1o20'W and 2°1'E, and acts as a transit point between the northern and southern parts of the nation”. 35 The projected population of the municipality based on the 2010 population and housing census is 118965 people, with 58,935 (49.5%) being males and 60,030 (50.5%) being females.^36^

### Study design

In this research, an analytical cross-sectional study design was used. The study design employed the quantitative approach to obtain information from participants.

### Study population

The study population comprises all healthcare professionals within the Kintampo North Municipality of Ghana.

### Sampling and sample size determination

The study's sample size was estimated using the Survey Monkey Sample Size Calculator.^37^

The estimated population of health professionals in Kintampo North Municipality was 736 as of January 2021. Using a confidence level of 95% and a 5% margin of error, the sample size was estimated to be 253 health professionals. The list of all 736 healthcare professionals was obtained from the Kintampo North Municipal Health Directorate. Using Microsoft Excel random numbers, the list was randomised and used for simple random selection of the target sample. The first 253 on the list were then recruited into the study without replacement.

### Data collection techniques and tools

The data was collected using a standardised self-administered questionnaire. The questionnaire was derived from some previously published studies 29,30,38 and modified. The survey was divided into three parts (Sections A, B, and C). Items on sociodemographic variables are found in Section A, perceptions of the COVID-19 pandemic and vaccines are found in Section B, and acceptability, hesitation, and determinants of COVID-19 vaccination are found in Section C.

The contacts of healthcare professionals in the municipality were collected from the Municipal Health Directorate (MHD). They were randomly contacted by phone to determine their willingness to participate in the study. Following consent, an electronic link was provided to health personnel through email and WhatsApp to complete the questionnaire. The electronic questionnaire was structured so that the same device (mobile phone or computer) could not submit the questionnaire twice, ensuring that such persons did not attempt the quiz more than once. Secondly, health professionals who do not have a WhatsApp number were contacted directly via phone to complete the questionnaire items. Those who consented to participate in the research and requested a hard copy questionnaire were printed and delivered to them to fill out and return in a sealed envelope. The data collection lasted 10 weeks, from January 2021 to March 2021.

### Data Analysis and Presentation of Results

Out of the 253 questionnaires distributed, 239 respondents returned filled questionnaires, representing a 94.0% response rate. However, after all questionnaires were audited for their completeness and signing of the consent form, 215 questionnaires were considered for analysis.

The Statistical Package for Social sciences (SPSS) software version 25 was used to code and analyse the data. The results were presented in tables. Chi-square analysis was utilised to compare categorical variables, and a p-value of 0.05 was considered statistically significant.

To determine the factors impacting COVID-19 vaccination uptake, a chi-square analysis was done.

A binary logistic regression model included all variables with P values of ≤ 0.25 in the chi-square analysis. The benchmark P value of ≤0.25 was selected because of its closeness to zero (0), thus enabling the inclusion of all potentially relevant predictive variables in the adjusted model. The fully adjusted model was fitted to assess how each independent variable affected the dependent variable.

### Ethical considerations

The Committee on Human Research Publications & Ethics, Kwame Nkrumah University of Science and Technology (KNUST) - Komfo Anokye Teaching Hospital (KATH), Ghana, gave clearance for this study with the reference ID: CHRPE/AP/225/21. Before being included in the study, each subject gave verbal and written consent. Subjects who refused to consent were not allowed to participate in the study.

## Results

### Socio-demographic Characteristics

With a mean age of 30.9 years and a standard deviation of 5.6, most respondents were in their 30s or older. Males formed the majority (56.3%) of respondents, while 40.9% of the respondents had a university first degree as the highest level of education. The majority (50.2%) of the HCP were married, 62.8% were Christians, and a higher proportion (40.5%) earned above GHS 2,000.00 (350 USD) as an average monthly income (Table 1).

**Table 1:** Socio-demographic characteristics of the study participants (N=215)

Variables	Category	Number (%)
**Age**	Less than 30 years	102 (47.4)
	30 years and above	113 (52.6)
	Mean±SD	30.9 ± 5.6
**Gender**	Male	121 (56.3)
	Female	94 (43.7)
**Highest level of education**		
	Certificate	44 (20.5)
	Diploma	54 (25.1)
	Degree	88 (40.9)
	Masters	29 (13.5)
**Marital Status**	Single	103 (47.9)
	Married	108 (50.2)
	Widowed/divorced	4 (1.9)
**Religion**	Christian	135(62.8)
	Muslim	80(37.2)
**Average Monthly Income**		
	Less than 1000	47 (21.9)
	Between 1000–2000	81 (37.7)
	Over 2000	87 (40.5)

### COVID-19 Perception, impact, and Vaccine intake history

Almost all respondents (96.7%) believed they had good health, and 98.6% believed COVID-19 cases were present in Ghana. More than half (54.9%) of the respondents believe that the risk of COVID-19 was low. The majority (86.5%, 90.2%, and 67.4%) of the study participants said COVID-19 impacts daily life, work, and income, respectively. Over 76% of the respondents knew someone who has taken the COVID-19 vaccine in Ghana and other countries, 45.1% had taken the influenza vaccine in the past, 30.2% knew the efficacy of the COVID-19 vaccine in Ghana, 7% had refused uptake of vaccines in the past, and the majority (57.7%) of respondents thought the COVID-19 vaccines in Ghana were safe (See Table 2 for details)

**Table 2:** COVID -19 perception, impact and vaccine intake history (N=215)

Variables	Category	Number (%)
**How do you see your health**		
	Good Health	208 (96.7)
	Poor Health	7 (3.3)
**COVID-19 cases confirmed in Ghana**		
	Yes	212 (98.6)
	No	3 (1.4)
**Perceived Risk of COVID-19**		
	High	97 (45.1)
	Low	118 (54.9)
**COVID-19 impacts daily lives**		
	Yes	186 (86.5)
	No	29 (13.5)
**COVID-19 impacts work**		
	Yes	194 (90.2)
	No	21 (9.8)
**COVID-19 impacts income**		
	Yes	145 (67.4)
	No	70 (32.6)
**Know someone who has taken the COVID-19 vaccine**		
	Yes	164 (76.3)
	No	51 (23.7)
**Have taken vaccine in the past**		
	Yes	97 (45.1)
	No	71 (33.0)
	Not Sure	47 (21.9)
**Have refused vaccine in the past**		
	Yes	15 (7.0)
	No	200 (93.0)
**COVID-19 vaccine is safe**		
	Yes	124 (57.7)
	No	91 (42.3)
**Knew efficacy of the COVID-19 vaccine in Ghana**		
	Yes	65 (30.2)
	No	150 (69.8)

### COVID-19 vaccine acceptance and preferences

Only 11.2% believed that vaccines alone could prevent COVID-19, while 68.4% believed that vaccines in general, were effective in preventing COVID-19.

The COVID-19 vaccine acceptance rate was 78.6% among HCPs, while 21.4% were hesitant to receive the vaccine. The findings of this study further revealed that 53.0% needed a medical officer's advice before they would take the vaccine, and 47.4% believed that vaccine convenience (i.e., place of vaccination, distance to the vaccine site, cost of the vaccine, etc.) would influence their decision to accept the COVID-19 vaccine. A little over half (51.2%) would be willing to take the vaccine as soon as it becomes available, while 48.8% opined that they would delay receiving the vaccine to monitor its safety among those who have taken it. Most (63.3%) did not prefer the origin of the vaccine (i.e., they were willing to take both imported and domestic). (Table 3)

**Table 3:** COVID-19 vaccine acceptance and preferences (N=215)

Variables	Category	Number (%)
**Would Vaccines alone prevent COVID-19**		
	Yes	24 (11.2)
	No	191 (88.8)
**Vaccine effective in Preventing COVID-19**		
	Yes	147 (68.4)
	No	68 (31.6)
**Willingness to accept vaccine**		
	Yes	169 (78.6)
	No	46 (21.4)
**Needs doctor's advice to take vaccine**		
	Yes	114 (53.0)
	No	101 (47.0)
**Convenience influence vaccine acceptance**		
	Yes	102 (47.4)
	No	113 (52.6)
**Readiness to take vaccine if available**		
	As soon as possible	110 (51.2)
	Delay, until confirmation of the safety of the vaccine	105 (48.8)
**Vaccine preference**		
	Imported	38 (17.7)
	Domestic	41 (19.1)
	Both	136 (63.3)

### Factors affecting the COVID-19 vaccine uptake

Table 4 shows factors affecting the acceptance of the COVID-19 vaccine. The study revealed that health professionals aged 30 and older were 2.2 times more likely to accept the COVID-19 vaccine (aOR; 2.2, 95 per cent CI;0.9–5.4, p=0.09). Also, respondents who knew someone who had received the COVID-19 vaccine were 14.9 times more likely to accept the COVID-19 vaccine (AOR; 14.9, 95 per cent CI; 5.0–45.0, p0.001). Respondents who had never refused any vaccines in the past were 7.8 times more likely to accept COVID-19 vaccines. Health professionals who believe vaccines are effective in preventing COVID-19 transmission are 5.0 times more likely to accept the COVID-19 vaccine (aOR=5.0, 95 per cent CI;2.1–12.4, p<0.001).

**Table 4:** Factors affecting the COVID-19 vaccine uptake

Factors	Measures	aOR	95 % Confidence Interval (CI)		P-value
			Lower	Upper	
**Age**	<30 years	Ref*			
	≥30 years	2.2	0.9	5.4	0.09
**Knew someone who has taken the COVID-19 vaccine**					
	No	Ref*			
	Yes	14.9	5.0	45.0	<0.001
**Have refused vaccine in the past**					
	Yes	Ref*			
	No	7.8	1.6	37.8	0.01
**COVID_19 vaccine is safe**					
	No	Ref*			
	Yes	9.2	3.3	25.8	<0.001
**Vaccine effective in Preventing COVID-19**					
	No				
	Yes	5.0	2.1	12.4	<0.001
**Perceived risk of COVID-19**					
	Low				
	High	1.8	0.7	4.6	0.2

Ref*-Reference, aOR-Adjusted Odd Ratios

Respondents who believed they were at high risk of COVID-19 were 1.8 times more likely to accept the COVID-19 vaccine (aOR=2.3, 95 per cent CI;0.7–4.6, p=0.2).

## Discussion

There exists a consensus among several researchers indicating the effectiveness of the COVID-19 vaccine against the spread of infection through the attainment of herd immunity.^39^,^40^ Nonetheless, the tendency to refuse the COVID-19 vaccine by the public has become an issue of global importance.^41^ Previous studies conducted else-where have given the varying acceptance of the COVID-19 vaccine.^22^,^41^–^43^

Most of the respondents indicated they were in good health. Respondents equally indicated that COVID-19 was present in Ghana but thought that they stood at a lower risk of getting infected. The respondents' perception of their health status and risk may not affect their readiness to accept the COVID-19 vaccine. The acceptance rate of the COVID-19 vaccine found in the present study was higher than reported by Agyekum et al. 21 (39.3%) and Fakonti 42 (30%) in related studies in Ghana and Italy among HCPs, respectively. Other studies reported a lower COVID-19 vaccine acceptance rate than the present study. For example, 63% was reported by Kwok et al. 44 (63%) and 48.6% in Hong Kong by Verger et al. 28 among HCPs. Adeniyi et al.^41^ reported a higher (90.1%) acceptance rate among HCPs compared to the present study in South Africa and a 91.3% acceptance rate among the Chinese population.^38^ There exist varying acceptance rates of COVID-19 vaccines across many regions of the world. Appreciating the holdups and facilitators, especially among HCPs who are highly at risk per the nature of their work for vaccine uptake, is therefore important. In our study, most respondents indicated that COVID-19 had impacted daily life activities, work, and income. Moreover, it was revealed by this study that about half of the HCPs would delay in taking the COVID-19 vaccine.

This is consistent with Wang et al.^38^, where 47.8% of the population was hesitant to accept the vaccine immediately. This position could be related to the widespread conspiracy theories about the COVID-19 vaccine, including vaccines containing infertility agent^45^, and political ideologies.^46^ Belief in these COVID-related conspiracy theories adds to vaccine hesitancy and, as a result, makes individuals mistrust the virus's preventive protocols and future vaccine acceptance.^47^–^49^ To limit the COVID-19 virus, it is critical to battle conspiracy theories and vaccination disinformation.^45^,^46^ Concerning the above, our study respondents explained that a medical officer's advice would encourage their uptake of the vaccine combined with other determinants such as convenience (where convenience denotes the place of vaccination, distance to the vaccine site, and cost of the vaccine). Adeniyi et al.^41^ also acknowledged positive perceptions, including direct contact with COVID-19 victims and the importance of the vaccine to end the pandemic combined with the vaccine's safety. These positive perceptions, as elucidated by respondents, may have influenced their readiness to accept the COVID-19 vaccine. The indication of medical officers' advice influencing respondents' vaccine uptake stresses the importance of health education programmes highlighting the vaccine's potential in controlling the spread of COVID-19 among the population. Other studies, including Verger et al.^28^, Agyekum et al.^21^ and Fakonti et al.^42^ highlighted that barriers to COVID-19 vaccine acceptance should all be considered in dealing with the challenge of vaccine hesitancy among HCPs.

Factors including age 30+ years, knowledge of someone who had taken the vaccine, those who had never refused a vaccine previously, the safety of the COVID-19 vaccine, the effectiveness of the vaccine in controlling COVID-19 transmission, and perceived higher risk of COVID-19 were good predictors of COVID-19 vaccine acceptance among respondents in our study. Elhadi et al. 50 reported higher odds of COVID-19 vaccine intake among persons aged 30+ years. Similarly, Fakonti et al.^42^ and Mesele^43^ reported higher odds of accepting the COVID-19 vaccine among HCPs and individuals who have received vaccines in the past. Reiter et al.^51^ found perceived higher risk and effectiveness as good indicators for COVID-19 vaccine acceptance among HCPs. The findings above are very much inconsistent with the findings of the present study.

The observed predictors in our study and others elsewhere 42,43,50,51 could be used to draw baseline targeted interventions and public health educational programmes. For example, individuals who have been previously vaccinated can be targeted among HCPs and at the community or population level to be vaccinated and used as educators to influence others to get vaccinated against COVID-19.

Again, as explained in some health behaviour change models 52, including the health belief model (HBM), perceived risk and severity have the tendency to cause individuals to adopt good health-seeking behaviours. The perceived risk as a good predictor for COVID-19 vaccine intake identified in our study could be used as a trump card to educate HCPs as well as the public on the threat including the severity of COVID-19 and hence the need to get vaccinated. Similarly, highlighting the safety of the vaccine could help increase vaccine confidence and acceptance among HCPs and the public.

### Limitations

Our study is a cross-sectional survey, representing a snapshot of HCPs' readiness to accept or not to accept the COVID-19 vaccine within the study's time frame stressing that the responses of the respondents are subject to change depending on factors, including additional vaccine safety data. Therefore, relying on the findings solely should be done with caution. The 215 participants studied, as opposed to the 253, which was the estimated minimum sample required for the study, could affect both the internal and external validity of the study. However, the evidence available shows that for a survey to be representative of the sample population, a response rate of ≥ 80% is expected.53 Also, the target response rate of 70% is considered the minimum for the generalisation of a survey.^54^ The response rate in this study was 94.0% as such, both the internal and external validity could not be compromised. Importantly, acknowledging similar findings elsewhere among recently published research supports our study in designing targeted public health interventions.

## Conclusion

This study indicated high COVID-19 vaccination acceptability among HCPs. However, some HCPs are hesitant to take COVID-19 vaccinations immediately. Factors such as knowing someone who had received the vaccine, acceptance of vaccines in the past, and safety and effectiveness of the COVID-19 vaccine determined vaccine acceptance. Increasing acceptance of COVID-19 vaccinations among HCPs and the Ghanaian population, in general, requires concerted efforts. This could be done by strengthening public health education on the perceived risks and safety of COVID-19 vaccines.
